# History of Tuberculosis

**Published:** 1884-10

**Authors:** Albert Johne


### ART. XXV.—THE HISTORY OF TUBERCULOSIS.

BY DR. ALBERT JOHNE.

(Continued from page 58.)

Frenzel wrote upon this disease in 1799, but was unable to free himself from the views of preceding authors; he also speaks of such animals as "fat and lean Franzosen"; the latter always being connected with an extreme degree of cachexia. The meat of such animals was looked upon as being exceedingly injurious to human beings. He considered the disease to be contagious, transmissible, and when fully developed, to be comparable to venerée in man. Augmented sexual desires occasion disturbances of the sexual organs, from which the *materia peccans* is conducted to other organs.

Veith considered the large nodular neoplasms to be due to luxurious proliferation in the elements of the serous membranes, on account of the preponderance or accumulation of coagulating lymph. Dietrich adopted the same views; but Gurlt considered the nodes to be of a tuberculous nature, but later adopted the idea that they were more of a sarcomatous character. D'Arboval looked upon the diseases as essentially of a phthisis nature, and described both forms as P. pulmonalis; while Hayne considered the neoplasms to be developed out of a formless lymphatic coagulum in consequence of rheumatic inflammatory processes in the serous membranes. Rychner and Thurm both considered nymphomania as an idiopathic complication, having nothing to do with tuberculosis. Hering and Fuchs were decided advocates of the tuberculous nature of the disease, the latter earnestly urging the connection between the Perl disease and pulmonary tuberculosis. Aside from the Perl disease, tuberculosis and scrofulosis of inner organs—that is, the mesenterial and bronchial glands—are also to be observed. Schellhose observed extensive tubercular degeneration in the larynx and trachea at the same time that the serosae were the seat of very diffuse perlzucht invasions. König, Anacker, and Kreutzer adopted similar views; while Dietrich looked upon the neoplasms as of exudative origin. Wolf considered them to be of a cystoid nature, strongly resembling artheromatous and colloid sacs.

The previous cited work of Virchow appeared at this time of strife and divergence in views among veterinarians. He emphasized the connection between the neoplasms found upon the serosae and within the organs, and considered them as tuberculoid, but at the same time of the character of lymphosarcomas, and contradicted decidedly any identity between them and tubercles in man.

But very few of the later veterinary authors have adopted the views of Virchow; of those most strongly opposed to these views we may mention Spinola and Humbold, who each defended the tuberculous nature of both processes. Gerlach, however, exceeded both these authors in his advocacy of the latter opinion; he did not look upon the results of the microscopic study of the tumors as decisive as to their nature, but considered that due consideration should also be given to the clinical and microscopical phenomena of the disease. He says: It is a fact—

1st. That in connection with the presence of noduli on the serosae we can always find tuberculous degeneration of the lymph glands, and, as a rule, also tubercles and cassations in the lungs as well as in other organs.

2d. That also in pulmonary tuberculosis, when in the vicinity of the pleura, noduli and nodes (trauben) may also be seen exactly as we find them in perlzucht, when the disease has extended to the pulmonary pleura.

3d. The perlzucht is hereditary, as is tuberculosis; the offspring from animals diseased with perlzucht may be tuberculous and vice versa.

Leisering assumed an intermediate position between the views of Gurlt, Virchow, and Rosell, and those of Gerlach, Spinola, Forster, etc. He considered that the neoplasms of perlzucht and tuberculosis all had their genesis in a newly developed and very vascular connective tissue, through secondary proliferation of its elements, their form being dependent upon the rapidity of the growth and proximity to one another, some being isolated, and others coalescing, forming the larger nodes. A course of rapid degeneration, either calcification or cassation, is common to them all. Simple softening only occur in those neoplasms which are situated in the submucosa of the respiration and digestive tracts, and the uterus; from these frequently occur ulcerative centres.

If, in our discussion of this question, we confine ourselves to the genetic standpoint, then we must look upon the neoplasms as of a sarcomatous nature; but if we look upon them in the condition of mature development, then their tuberculous character becomes more prominent. Although they cannot, so it seems, be forcibly brought directly into either class, yet they must be looked upon as a neoplasm *sui generis* peculiar to cattle, sometimes swine, and can therefore be designated as bovine tuberculosis.

### THE PERIOD OF EXPERIMENTAL INVESTIGATION INTO THE NATURE OF TUBERCULOSIS, BEGINNING WITH VILLEMIN.

The tuberculosis question entered upon an entirely new phase of life when Villemin (1865) declared it to be a specific disease, transmissible from man to animals, and among the latter from one species to another. No etiological or pathological question in the history of medical development has ever been subjected to more skeptical criticism and observation than the one introduced by Villemin. Experiments by hundreds and thousands have been made, as well as the most exacting histological studies, in order to arrive at the true nature of this disease and its products. The results of all this endeavor have not been in vain, and gradually we see that order has developed out of an apparently obscure chaos, so that today we can scarcely find an authority who throws a doubt upon the infectio-contagious nature of tuberculosis and the identity of the disease, whether found occurring in man or animals.

### EXPERIMENTS.

All the experiments which have thus far been made with tuberculosis material, in order to induce the disease in any species of animal, have been either of an inoculative, inhalation, or feeding character.

### 1.—INOCULATIVE EXPERIMENTS.

Innumerable experiments of almost every form and variety have been made in this direction. Tuberculosis material from men and animals has been introduced under the skin, in the abdominal cavity, in the larger blood vessels, or in the anterior chamber of the eye, or even into the lungs themselves by direct operative interference.

Subcutaneous inoculations occupy the first place among these experiments. Toussaint concludes, from those made by him, that no disease is more infectious than tuberculosis, and that all the fluids of the body, the blood, nasal secretion, saliva, the juices of the tissues, and the urine of tuberculous animals, even the contents of the vesicles of inoculated variola, are all conveyors of the infectious elements. The latter retain their activity in a temperature which cannot be borne by anthrax bacilli. These experiments have been made upon cows, calves, goats, swine, rabbits, and dogs, and almost invariably have led to the development of miliary tuberculosis. Nearly all the authorities, Virchow excepted, admit the identity in construction of these tubercles with those in man.

Opposed to the great number of positive results following these inoculations, we have a surprisingly small number of a negative character. Of the latter are those of Putz, two calves inoculated with tuberculosis material from man; Köhne, three horses with material from cattle, and fresh pus from horses; Verga, two mules, one cow, two sheep, and two dogs; Semmer, dogs and horses with tuberculous material from cows, etc. Experiment has shown that the solipeds and carnivora possess an almost absolute degree of immunity, or at least are very insusceptible to infection, in comparison to the ruminants and other vegetarian animals, of which the rabbits and guinea pigs are remarkably susceptible.

The introduction of tuberculous material into the peritoneal cavity has been tried, but to no such extent as the previous manner of experiment. Klebs, Bollinger, and Kitt have been noteworthy. Klebs succeeded in producing tuberculosis of the peritoneum in a calf, that presented the same phenomena as perlzucht, with material derived from man. Bollinger attained the same results in a goat by injecting substance taken from a tuberculous-affected lymph gland. Kitt had similar results from a calf, using also material taken from man.

The results of these various experiments have not, however, been adopted without some degree of skepticism. A great number of investigators have endeavored to prove that the inoculation of non-tuberculous animal derivatives, or even the introduction of various indifferent substances, especially in rabbits and guinea pigs, has been followed by the formation of caseous centres, and in some cases by miliary tuberculosis. Waldenberg declared the tuberculosis resulting from inoculation to be non-specific in its character, being due to the absorption of fine particles of the substances introduced, and their transportation to different parts of the organism by means of the circulation, and their accumulating acted as foreign bodies, causing local neoplastic centres. Tuberculosis can be generated not only by foreign elements thus introduced, but auto-infection can also take place from caseous or suppurative centres in the body, which have nothing specific about them—a theory which conforms, in a measure, to the caseo-infection theory of Buhl. Friedlander went so far as to declare the inoculative tuberculosis to be an illusion, and the experimental generation of the disease as impossible.

These assertions, and the negative results of many experimenters, were not influential enough to overcome the value of the testimony gathered from positive experiments.

Ponfich's, Hoffman's, and Langerlan's experiments with Zinnaher in the peritoneal cavity of guinea pigs, entirely affect the assertions of Waldenberg, for tuberculosis did not follow in any of them. In cases where the inoculation of non-tuberculosis materials have even been followed by the development of tuberculoid eruptions, and although such neoplasms almost corresponded with those of tuberculosis from an histological point of view, still their further deportment plainly demonstrated the absence of non-specific characteristics—that is, they do not undergo caseation, nor were they suitable for further inoculation. Toussaint declares the transmissibility of true tuberculosis to be unlimited ; he even asserts that the virulence of the inficiens increases in progressive inoculations. Martin came to the same results ; he calls the tuberculoid productions following the embodiment of non-specific materials, pseudo-tubercles, though he admits their histological correspondence. The only point of differentiation is the power of secondary inoculation or auto-infection.

Other experimenters have demonstrated that the inoculation of non-tuberculous substances—flesh, neoplasms of other kinds, foreign bodies, etc.—are not followed by the formation of tuberculoid or caseous centres at the point of inoculation in all animals, nor even in all members of so-called susceptible species. Rabbits, by which a tendency to caseation is very common, may also be classed under this head. On the contrary, from the inoculation of genuine tuberculous material, infection has resulted in species in which it could not be produced in any other way. We can safely say that such results are much more surely to follow inoculations made with specific than with non-specific material, even in susceptible animals.

It was especially important to show that not every cheesy mass was capable of producing tuberculosis *per* inoculation, but that in general, when it did follow such inoculations, the material used, or the wound, was in some way contaminated with tuberculous virus.

Finally, it has been many times demonstrated that the generation of a caseous centre at the point of inoculation is not necessarily followed by the development of tuberculosis.

These various experiments served to confirm, more and more, the hypothesis of Villemin of the infectiousness and unity of the tuberculosis of man and animals.

*Intravascular inoculations* have been conducted by Semmer, Thal, Nesserow, Spinola, Colin, *et al.*, upon swine and sheep. Thirty animals were inoculated with milk and blood taken from a cow that was very far advanced in both pulmonary and pleural tuberculosis ; an amount equal to one-twelfth or one-fortieth of the total quantity of blood in the animal inoculated was introduced into the jugular. In no case did suppuration or caseation occur at the point of inoculation. Thirteen of the animals died from accidental causes; sixteen lived, and were killed at the end of five and six months. In the pigs, the tuberculous processes exactly resembled those found in the lungs and pleura of cattle; while in the sheep they resembled those seen in man. Basing his conclusions on these experiments, Semmer says that "the perlzucht of cattle is transmissible to swine, and that the inficiens is found in the blood, flesh, and milk, and that sheep and other animals are also susceptible to infection." In opposition to Gerlach, Klebs, Orth, Schuppel, *et al.*, he considers the perlzucht of cattle to be an idiopathic, and therefore not a disease completely identical with human tuberculosis.

Putz introduced tuberculous material from man into the lungs of two horses; in one case the result was questionable, but in the other a typical miliary tuberculosis resulted.

*Intraocular inoculations* were at first made by Cohnheim and Salmonsen, and soon followed by those of Hänsell and Deutschmann, in order to test the virulence of human tuberculosis, were afterwards repeated by Baumgarten with reference to bovine tuberculosis. These experiments were made with the most scientific exactness; fresh, non-casefied tuberculous material was introduced into the anterior chamber of the eye, with result of exciting a typical iris-tuberculosis, and in general, also, a deutropathic, general tubercular invasion of the organism. The experiments of Baumgarten with bovine material gave not only the same ocular results, but were followed by more profuse generalization than those made with human material. These experiments proved beyond all question that *only pure tuberculous material from either man or animals can produce unquestionable results.*

### 2.—INHALATION EXPERIMENTS.

These experiments were made with tuberculous material finely suspended in water, and with proper instruments, either as a spray, introduced into the trachea, or the air which the animals breathed was saturated with the same material. Nearly all such experiments have been followed by positive results, and go to form not only the specific virulency of tuberculosis, but also the possibility of the aspiration of the infectious elements by the respiratory tract, and the dangers of cohabitation,

### 3.—FEEDING EXPERIMENTS.

Inhalation and inoculative experiments have sufficiently proven the general virulence and identity of tuberculosis of man and cattle.

Another question of vital importance that soon interested experimental pathologists, was whether the consumption of the milk and flesh from cattle diseased with tuberculosis could cause the disease in other animals, and eventually man. The first investigations in this direction were made by Gerlach (1866–69) with the tuberculous nodes from the serous membranes of cattle, and with the milk from a cow diseased with perlzucht; while Chauveau's experiments were made in 1869. Gerlach and Chauveau declared tuberculosis to be an eminently infectious disease, basing their assertions upon these experiments. Subsequently a great number of the most eminent pathologists busied themselves in experiments of this nature. But a very small number of these men have recorded negative, while some of them report almost exclusively positive results.

We have a record of 322 feeding experiments made upon various kinds of domestic animals with raw material. Of these, 47.7 per cent. gave positive, and 48.9 per cent. negative results, and 3.3 per cent. results of a doubtful character. In sixty-two experiments the material used was cooked for from ten to fifteen minutes; these gave 35.5 per cent. positive and 64.5 per cent. negative, and 1.6 per cent. doubtful results. (For the exact details of each of these experiments, the reader is referred to the original paper.)

To the positive results from these experiments we have to add a number of clinical observations from Jakobs, Devilliers, and Langer with reference to the transmission of tuberculosis to dogs and hens by means of their eating the sputa from phthisical human beings, as well as those reported by Klebs, Goring, Zippelius, Lehnert, Kloss, and Bottcher, with reference to the transmission of tuberculosis from diseased cattle to calves and swine by means of the milk or flesh of such cattle. These results lead to the following conclusions:

1st. The transmission of tuberculosis from animals to animals, and from man to animals, by means of the consumption of tuberculous material, though the results are less certain than those to be expected from inoculation.

2d. Transmission occurs more readily from feeding tuberculous material, than from the use of milk derived from tuberculous diseased organisms.

3d. Transmission does not so readily occur (1.6 per cent. of all the experiments) when the flesh from such animals is alone used.

4th. Calves, sheep, goats, and swine possess the greatest degree of susceptibility, as has been shown by Bollinger; while the carnivora possess a very slight degree.

(Of 100 carnivora fed with such material by Semmer, none became infected.)

5th. It would appear as if the same kind of material taken from the same species of animals did not always have the same degree of infectiousness.

(*a*) This variation is also to be traced to a varying degree of susceptibility among the animals used for experiment. Young animals are more susceptible to infection than older ones.

(*b*) With reference to the infectiousness of milk, it is undoubtedly a fact that it is of importance whether the udder has or has not become the seat of tuberculous invasion.

(*c*) The duration of time during which such experiments are continued is undoubtedly of importance. Bollinger, Orth, and Semmer have called attention to this circumstance with reference to the negative results of many experiments, on account of the protracted development of tuberculosis. From their own observations, they conclude that at least two to three months' continued feeding is necessary before one should conclude that infection was impossible.

(*d*) With reference to the experimental feeding of milk, we should not lose sight of the difficulties surrounding the intra-vital diagnosis of tuberculosis in the cows from which such milk is derived. Such cows should always be subjected to a microscopical examination before definite results, as to the experiments, are recorded.

6th. We should never lose sight of the true value which even a few positive results bear in comparison to many negative in the field of experimental pathology.

### 4.—HISTOLOGICAL EXAMINATIONS.

That the previously detailed experiments should at once excite the most exacting histological examinations of their results, as well as accurate comparison between them and the tubercle of man and animals, is not at all surprising. The definition of a tubercle originally given by Virchow is still adhered to in its general characteristics; i. e., a neoplasm of nodular form, cellular construction, non-vascular, that later on undergoes a process of cheesy degeneration. Later investigations have, however, shown that the lymphoid cells, which enter largely into the construction of a tubercle, are not its only cellular element, but that other cells also take part in its development, and help largely to give to it its peculiar characteristics. The giant-cells first described by Rokitansky, and afterwards by Laughonus *et al.*, play a very important rôle in the natural history of a tubercle. Schuppel not only speaks of their constancy as a tubercle phenomenon, but considers them also to be the centrum, or nestrix, from which the other cells develop. According to his investigations, these cells are surrounded by large epitheloid cells, while the lymphoid cells occupy the peripheries of the tubercle. According to Wagner, these cellular elements are interlaced by a delicate reticulum of connective tissues. Koster and Charcot also look upon giant cells as a constant element in the construction of a tubercle; while many other authorities speak of them as having a pathognomonic value in diagnosis; others, however, of equal eminence, absolutely deny all value to this last assertion. Hering denies all specific importance to the giant or epitheloid cells, and supports his assertion by quoting their frequent absence in tubercles.

Later studies of this question have shown that tubercles, without the characteristics demanded by Schuppel, and having those described so accurately by Virchow, are developed in immediate proximity to the former. Giant cells have also been found in the most varied pathological neoplasms, which have no connection whatever with tuberculosis. Ziegler does not look upon either the giant or epitheloid cells as anything peculiar to tuberculosis, as they are not limited in their occurrence to these neoplasms. According to him, the cells found in tubercles entirely correspond to those that occur in granulation processes. All modern investigators are united in looking upon the tubercle as a neoplasm of inflammatory origin, occurring in the same way as ordinary granulations, and composed chiefly of migrated white blood cells—leucocytes—while the endothelium of the lymphatics and the fixed connective cells assume a very unimportant part in their development. The difference between granulations and tubercles is that the multinuclear cells are much less frequently seen in the former than in the latter. While the cells that take part in granulation gradually lead to the construction of connective tissue, those of the tubercle do not lead to any such maturity, but rapidly undergo retrogressive metamorphosis—caseation. Pflug speaks of actinomycosis tubercles, as he found in the centre of nodular neoplasms, having every microscopic appearance of tubercles, the peculiar fungi of this disease, and therefore considers that all nodular formations should be known as tubercles, irrespective of their slight anatomical differentiations, their true character being indicated by adjectives, i. e., T. phthisicum, etc.

Parallel with the study of the tubercle in man, proceeded that of similar formations in animals. As already indicated, as great a divergence of opinion ruled in this regard as with reference to the tubercle in man, and also as to the anatomical correspondence between the two neoplasms—a view of the question that has been adopted by the majority of the authorities, not without some objections, however. Virchow has even lately placed especial emphasis upon the fact that the tubercle in cattle is not subject to caseation, but is, on the contrary, to general calcification; that is, varies in form from the tubercle in man, and also in its appearance as groups or collections upon the serosae, and that these variations are of a sufficient character to nullify the identity of the bovine and human tubercle.

Günther, Hooms, *et al.*, have, however, directly contradicted these assertions, affirming that caseation does occur in the bovine tubercle, and that the nature and conditions of the supply of nutriment is the determining cause as to whether caseation or calcification results in the bovine tubercle. In young animals the tendency to caseation predominates.

Baumgarten especially emphasizes the fact that caseation occurs identical with that occurring in the tubercle in man, and that it can be easily demonstrated by careful decalcification of such tubercles. Lwow also takes sides against Virchow with reference to the calcine-metamorphosis in the bovine tubercle, declaring it not to be an essential characteristic, and also that caseous metamorphosis is not an absolute factum in human tuberculosis. The perlknoten (nodes) more strictly resemble chronic tuberculosis in man, as they largely consist of a confluence of several tubercles—Rudneff—and according to Virchow, consist largely of connective tissue, and also the nodes in perlzucht (zucht means a chronic disease). The differentiation between the microscopic appearance of the tubercles in the serosae in cattle and those in man, as decided by Virchow, is contradicted by the researches of Creighton, who describes eight cases of tuberculosis in man, in which he found flat and necklace-like ("perlschnurartig") tuberculous deposits upon the serosae.

As previously mentioned, Virchow considers the bovine tubercle to have lympho-sarcomatous characteristics, which leads Semmer to doubt the complete identity of these neoplasms in man and cattle. According to Semmer, the tubercle in cattle is of a more sarcomatous character, on account of the prevalence of connective tissue in its structure; while those occurring in sheep more exactly resemble those in man. These views are contradicted by Schuppel, *et al.*, on account of the fact that besides the fibrous variety, may also be seen typical cellular tubercles in the lung of affected cattle. Ziegler does not consider these variations to be of the slightest moment. Birsch-Hirschfeld considers these different conditions to represent simply various phases or stages in the development of the neoplasms, as well as individual idiosyncrasies. The weight of testimony and an unbiased judgment would seem to lead one to look upon these neoplasms as essentially identical in their construction.

Orth confirms this opinion by various experimental and histological researches of a most exact character.

Feeding experiments with the milk from cattle diseased with tuberculosis have led to very exhaustive studies of the tissues of the udder, as well as its secretion. Gluge (1850), and after him Bruckmüller and Fürstenberg, called the first attention to the presence of tubercles in the udder of cows. Later literature finds many records from various authors confirming these earlier investigations, and also of the connection between the tubercles developed in calves from the consumption of milk from such udders.

Kolessnikow looks upon the tubercles found in the udder as identical with those of perlzucht, adopting Virchow's views in this respect, under whose guidance his studies were made. Schuppel has, however, made a most exacting and critical study of this question, and emphatically declares these tubercles to be identical with those occurring in man. Virchow considers that tuberculosis of the udder in milch cows is a question of the most urgent importance, from a public health point of view. Chemical and physical investigations with reference to the nature of milk from cows thus affected, have thus far only led to no important results. Billardière found that the milk from such cows (poris) contained seven times as much phosphate of lime as that taken from healthy cows. Lupuy confirms this assertion. Lehman found the amount of casein diminished. Dutrone considered bluish-looking milk to be indicative of tuberculosis; while others could find no essential difference between the milk from such cows and that from healthy ones.

The improvement in the means and methods of histological research have, however, led to many interesting conclusions, and advanced still further our knowledge of the more delicate anatomical characteristics of the tubercle. Schuppel's views have found much confirmation; and neoplasms which were (Virchow) formerly looked upon as of inflammatory, hyperplastic, or scrofulous origin, have become classed as tubercles. In man, typical tubercles have been found in scrofulous lymphadenitis, in caseous pneumonia, and in certain forms of fungus-inflammation of the joints.

All these varied processes have this characteristic in common with known tuberculosis, viz: by inoculation upon animals they have produced both local and general tuberculosis; and notwithstanding certain variations in their microscopic appearances, they seem to have a common genesis.

(*To be continued.*)

